# Testing mechanical chest compression devices of different design for their suitability for prehospital patient transport - a simulator-based study

**DOI:** 10.1186/s12873-021-00409-3

**Published:** 2021-02-04

**Authors:** Maximilian Jörgens, Jürgen Königer, Karl-Georg Kanz, Torsten Birkholz, Heiko Hübner, Stephan Prückner, Bernhard Zwissler, Heiko Trentzsch

**Affiliations:** 1 Institut für Notfallmedizin und Medizinmanagement (INM), Klinikum der Universität München, LMU München, Schillerstr. 53, 80336 München, Germany; 2 LAKUMED Klinken - Krankenhaus Vilsbiburg, Klinik für Anästhesie und Intensivmedizin, Vilsbiburg; Ärztlicher Leiter Rettungsdienst (ÄLRD), Landshut District, Germany; 3 Klinikum rechts der Isar der Technischen Universität München, Klinik und Poliklinik für Unfallchirurgie, München; Ärztlicher Bezirksbeauftragter Rettungsdienst (ÄBRD) Northwest Upper Bavaria, München, Germany; 4 Universitätsklinikum Erlangen, Anästhesiologische Klinik, Erlangen; former Ärztlicher Leiter Rettungsdienst (ÄLRD), Amberg District, Germany; 5 Medical Director of Emergency Services, Zweckverband für Rettungsdienst und Feuerwehralarmierung Allgäu, Kempten, Germany; 6 Klinik für Anästhesiologie, Klinikum der Universität München, LMU München, Munich, Germany

**Keywords:** Cardio-pulmonary resuscitation, Mechanical chest compressions, Pre-hospital emergency medical services, Transport, Device stability

## Abstract

**Background:**

Mechanical chest compression (mCPR) offers advantages during transport under cardiopulmonary resuscitation. Little is known how devices of different design perform en-route. Aim of the study was to measure performance of mCPR devices of different construction-design during ground-based pre-hospital transport.

**Methods:**

We tested animax mono (AM), autopulse (AP), corpuls cpr (CC) and LUCAS2 (L2). The route had 6 stages (transport on soft stretcher or gurney involving a stairwell, trips with turntable ladder, rescue basket and ambulance including loading/unloading). Stationary mCPR with the respective device served as control. A four-person team carried an intubated and bag-ventilated mannequin under mCPR to assess device-stability (displacement, pressure point correctness), compliance with 2015 ERC guideline criteria for high-quality chest compressions (frequency, proportion of recommended pressure depth and compression-ventilation ratio) and user satisfaction (by standardized questionnaire).

**Results:**

All devices performed comparable to stationary use. Displacement rates ranged from 83% (AM) to 11% (L2). Two incorrect pressure points occurred over 15,962 compressions (0.013%). Guideline-compliant pressure depth was > 90% in all devices. Electrically powered devices showed constant frequencies while muscle-powered AM showed more variability (median 100/min, interquartile range 9). Although physical effort of AM use was comparable (median 4.0 vs. 4.5 on visual scale up to 10), participants preferred electrical devices.

**Conclusion:**

All devices showed good to very good performance although device-stability, guideline compliance and user satisfaction varied by design. Our results underline the importance to check stability and connection to patient under transport.

## Background

The use of mechanical chest compression (mCPR) devices does not provide an improved survival rate compared to manual chest compression [1–4]. However, there are situations in which mCPR appears to offer advantages and the guidelines of the European Resuscitation Council (ERC) consider mCPR devices as a “reasonable alternative” [5–7]. This is especially true in situations when ROSC is unlikely to occur as a result of a “stay-and-play”-approach and when more sophisticated interventions will be required e.g. catheter intervention in myocardial infarction with refractory ventricular fibrillation or prolonged efforts are necessary to eliminate the root causes of cardiac arrest e.g. in severe hypothermia or when antagonizing tricyclic antidepressant intoxication. However, protracted manual chest compression leads to a loss of quality during resuscitation and is negatively influenced under transport conditions [8–11].

There are already several simulator-based studies available that analyze how various mCPR devices perform during prehospital transport [12–18]. Some focused on transport in helicopters [12, 13] most looked into ground-based transport [14–18] and most of the times, only one single device was compared to manual CPR as standard intervention [13–17]. Gaessler et al. compared LUCAS2, Autopulse and animax mono during ground ambulance transport, but excluded all other settings during ground-based pre-hospital transport [18]. Dringhaus et al. evaluated various evacuation routes but were restricted to Corpouls CPR only [15]. To our best of knowledge, there is no study so far, that has compared mCPR devices of different design their performance within all relevant settings during ground-based prehospital transport. That includes of course ambulance transport including loading/undloading but also turntable ladder and transport through staircases. In order to draw up specifications for the requirements of mCPR equipment for centralized procurement in the German Free State of Bavaria, we carried out the study presented here.

The aim of this study was to evaluate mechanical chest compression devices in pre-hospital patient transport under resuscitation regarding potential construction-design differences under realistic conditions of use.

## Methods

### mCPR devices

Four devices were tested: animax mono (AAT Alber Antriebstechnik GmbH, Albstadt, Germany), AutoPulse reanimation system model 100 (ZOLL Medical Corporation, Chelmsford, Massachusetts, USA), corpuls cpr (GS Elektromedizinische Geräte G. Stemple GmbH, Kaufering, Germany) and LUCAS2 (Physio-Control, Inc., Redmond, Washington, USA). Animax mono is operated by muscle power. The other devices have an autonomous electric drive system. Animax mono, corpuls cpr and LUCAS2 have a stamp mechanism; AutoPulse employes a load distributing band (LDB) that compresses the thorax semi-circularly. The devices have been described in detail elsewhere [19, 20].

### Transport team

Nine paramedics and four emergency physicians participated in this study, building a four-person transport team to carry a mannequin under mCPR along a pre-defined route. The turntable ladder was operated by a crew from Munich Fire Department.

### Mannequin and equipment

The simulator (Ambu® Man W (Wireless); Ambu GmbH, Bad Nauheim, Germany) weighed 14 kg. We added 50 kg of lead pellets to increase the load. It features a realistic airway to facilitate all sorts of airway management. In this settig, the mannequin was intubated via orotracheal route. The endotracheal tube was secured with a Thomas Tube holder (Leardal Medical GmbH, Puchheim, Germany). In addition, the team had to carry a defibrillator (LIFEPAK® 15; Physio-Control, Inc., Redmond, Washington, USA) and a 2-l oxygen cylinder with pressure reducer. Transport teams were trained in proper use of the mCPR devices and worked under supervision by manufacturer representatives.

### Transport route, route stages and procedure

The 10 stages of the total transport route consisted of transport with a *soft stretcher* or *gurney involving* a staircase, vehicular trips with *turntable ladder* and rescue basket and *ambulance transport*, as well as *loading* and *unloading*.

During transport, a member of the transport team ventilated the mannequin using a respiratory bag in a compression-ventilation ratio of 30:2 (exceptions: *ambulance transport.* Continuous use of mCPR device and mechanical ventilation with MEDUMAT Standard (WEINMANN Emergency Medical Technology GmbH, Hamburg, Germany); *rescue basket* - ventilation with an Oxylator (CPR Medical Devices, Inc., Markham, Ontario, Canada)).

### Data collection

Resuscitation data was recorded using Ambu CPR Software (Version 3.1.1). Data were collected for each stage individually. Transport teams were blinded to the recordings.

### Endpoints

The study endpoints included stability of the device and compliance with guideline criteria for high-quality cardiac massage according to 2015 ERC guidelines.

For this purpose, compliance with the correct pressure point was analysed; for chest compression this point was on the lower half of the sternum for all mCPR devices with exception of AutoPulse [21]. Deviations in the mCPR devices’ connection position were measured in cranio-caudal or lateral direction using callipers and a scale burned into the skin of the mannequin. For AutoPulse, lateral displacement was defined as twisting of the LDB in the frontal plane. If dislocation occurred, devices were re-adjusted at the end of each stage. In addition, the number of stages with correction of the connection position was compared to those without.

Criteria for high-quality chest compression included compliance with a compression depth of 50–60 mm and a compression-ventilation ratio of 30:2. Rounded means of complete compression-ventilation cycles with “30:2” were classified as “OK” and “Not OK” if this was not the case.

The guideline recommended frequency of 100–120 chest compressions per minute can be guaranteed only by corpuls cpr and LUCAS2. Autopulse is set by the manufacturer to a frequency of 80 per minute; animax mono is user-dependant.

The transport team evaluated each mCPR device after each run on a standardized questionnaire using visual analogue scale from 0 (“totally unsuitable”) to 10 (“ideally suited”) in four categories and ranked the perceived physical effort using a modified BORG CR-10 scale (0 ≙ no exertion/breathlessness; 10 ≙ maximum exertion/breathlessness forces stop) [22]. Additionally, positive or negative aspects could be indicated in open text responses.

### Study design

The test sequence of the devices was carried out following a web-based block randomization [23]. Two passes were completed (second pass modified: no trip with *rescue basket*; one instead of two *turntable ladder* transports).

For statistical analysis, similar action sequences, such as basic resuscitation, loading and unloading of the ambulance, or all turntable ladder movements were grouped accordingly resulting in 6 groups that underwent statistical analysis. Stationary mCPR (*basic resuscitation)* at the beginning and end of the transport served as control group.

Data were checked for normal distribution using Shapiro-Wilk test. Numerical data is given as median with interquartile range (IQR) and categorical data as percentage (%). Unless otherwise specified, we used Fisher’s exact test for comparison of categorical data due to small sample size. Statistical tests were selected according to type of feature and type of scale level: *p* < 0.05 was considered a statistically significant difference.

The study protocol was reviewed and approved by the Ethics Committee at the Medical Faculty of the LMU Munich (EK Nr. 493–15). Members of the transport team gave consent to participate in the study.

## Results

### Device-stability under transport conditions

#### Correct pressure point

An incorrect pressure point was recorded for 2 of 15,962 compressions (0.013%). Both were measured at the beginning of the application of corpuls cpr during soft stretcher transport.

#### Pressure point displacement

Table 1 gives an overview of maximum displacements in cranio-caudal and lateral directions.

**Table 1:** Devices and their maximum displacements (cranio-caudal; lateral)

Device	Max. displacement (cm)	
	cranio-caudal	lateral
animax mono	4.5	1.0
AutoPulse	2.3	2.2
corpuls cpr	1.7	1.0
LUCAS 2	0.5	0.4

Comparing connection-point-disloction, animax mono is particularly striking. Cranio-caudal slippage of LUCAS2 occurred only during *basic resuscitation* and ambulance transport and was lower, with a maximum of 0.5 cm. AutoPulse’s LDB twisted through the frontal plane by up to 2.2 cm.

None of the detected displacements led to detection of an incorrect pressure point.

#### Frequency of correction after slipping

The total number of corrections after slippage was used to compare device-stability (Table 2).

**Table 2:** Comparison of corrections after each route stage (by device)

Device		Correction of pressure point required?	
		No	Yes
**animax mono**	Number of route stages observed	3	15
	**Percentage (%)**	**17**	**83**
**AutoPulse**	Number of route stages observed	10	8
	**Percentage (%)**	**56**	**44**
**corpuls cpr**	Number of route stages observed	9	9
	**Percentage (%)**	**50**	**50**
**LUCAS 2**	Number of route stages observed	16	2
	**Percentage (%)**	**89**	**11**

Pairwise comparison showed significant differences in the transport stability of AutoPulse (*p* = 0.04) and LUCAS2 (*p* < 0.001) compared to animax mono. In addition, LUCAS2 had to be corrected significantly less often than the corpuls cpr (*p* = 0.03).

### Proportion of compressions meeting guideline pressure depth

**Fig. 1:**
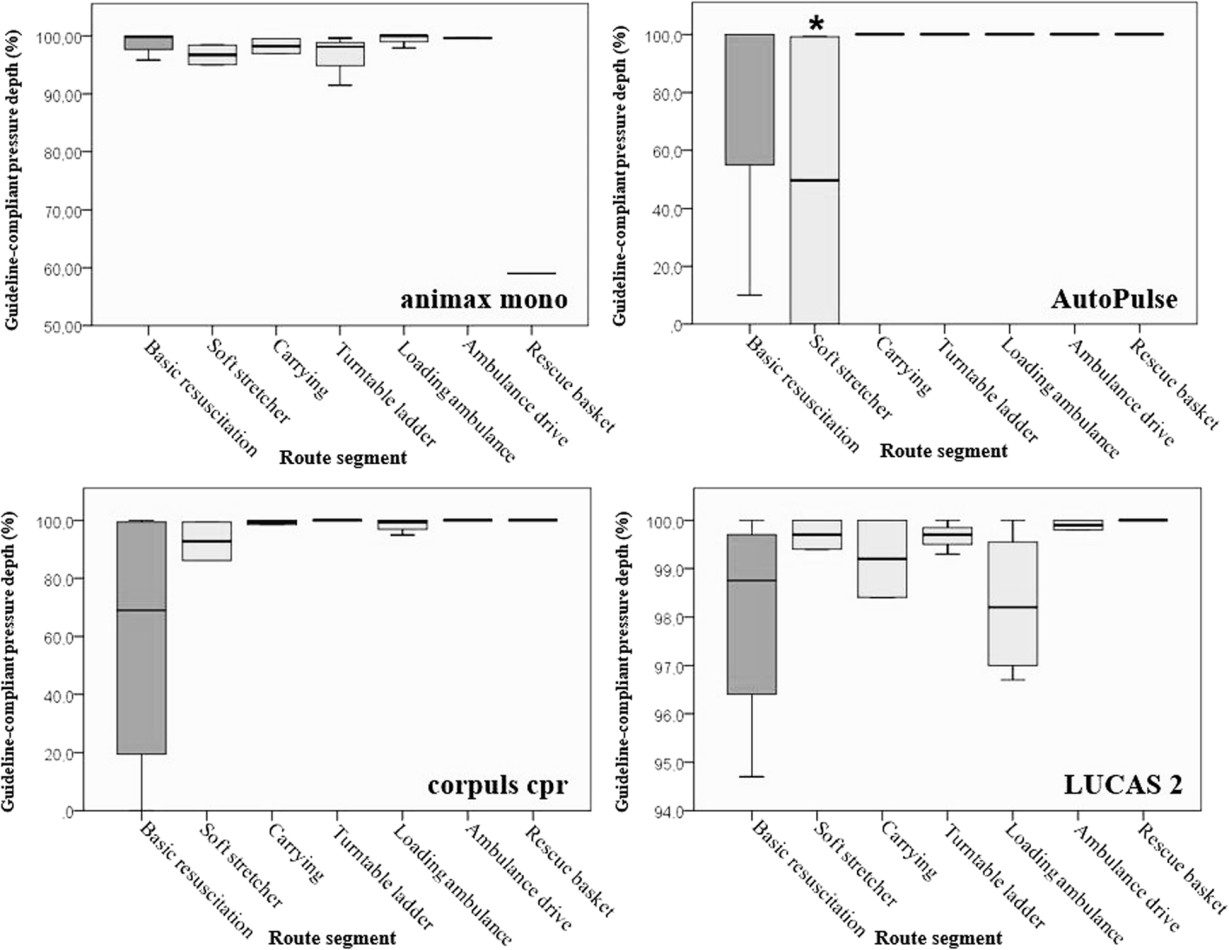
Proportion of compression with pressure depth meeting guidelines (%), by device and route. (*) Faulty data recording on the first run (− unintended termination after 20 s; limited information

The proportions of compressions with pressure depth meeting the guidelines under transport conditions were compared to *basic resuscitation* in stationary operation, separated by device and stage (Fig. 1).

Overall, compression performance for all devices was over 90% on most stages. One outlier was observed with animax mono during *rescue basket* transport, where 40.1% were too deep compressions.

With AutoPulse applied during *soft stretcher* transport, compression performance could be assessed only to a limited extent (49.6%) compared to other stages, because a data cable disconnected during the first run and interrupted recording (* in Fig. 1).

One conspicuous result was that with all electrically powered mCPR devices (AutoPulse, corpuls cpr and LUCAS2) greater scattering in the guideline-compliant pressure depth was observed during *basic resuscitation*.

### Frequency stability

Frequency for LUCAS2 (102/min), corpuls cpr (100/min) and AutoPulse (80/min) was consistently achieved (IQR 0). Compression rate for the muscle-powered animax mono showed greater variability (100/min, IQR 9).

### Compression-ventilation ratio

The devices were tested pair-wise for significant differences in the compression-ventilation ratio.

In the first run, data recording was interrupted during soft stretcher transport with AutoPulse (* in Table 3). As a result, *n* was reduced here from 15 to 14. Animax mono differed significantly from AutoPulse (*p* = 0.005), corpuls cpr (*p* = 0.02) and LUCAS2 (p = 0.02). The other devices performed similarly in the comparisons (data not shown).

**Table 3:** Comparison of the 30:2 compression-ventilation ratio after each route stage (by device)

Device		“30:2” ratio	
		Not OK	OK
animax mono	Number of route stages observed	9	6
	**Percentage (%)**	**60.0**	**40.0**
AutoPulse	Number of route stages observed*	1	13
	**Percentage (%)**	**7.1**	**92.9**
corpuls cpr	Number of route stages observed*	2	13
	**Percentage (%)**	**13.3**	**86.7**
LUCAS 2	Number of route stages observed	2	13
	**Percentage (%)**	**13.3**	**86.7**

### Assessment by the users

Study participants evaluated the devices in four categories (1 ≙ totally unsuitable; 10 ≙ ideally suited). Physical burden was assessed on the modified BORG scale.

Use of the electrically powered mCPR devices showed a high level of satisfaction, regardless of the category. The manually operated animax mono achieved worse values, although effort during transport was rated almost equally on the modified BORG scale.

With medians of 4.0 (AutoPulse, corpuls cpr IQR 3; LUCAS2 IQR 2) and 4.5 (animax mono IQR 3), respectively, perceived physical burden corresponded to a (marked) exertion, which *by definition* was accompanied by noticeable but controllable breathing. Analysis of satisfaction showed clear differences in the Kruskal-Wallis test (Table 4). When we rejected the nullhyopthesis, that there was no difference in the variance of the responses, we performed pairwise comparison of the various devices respectively. Results are in Table 5: While there was no significance between animax mono and AutoPulse or animax mono and corpuls cpr in turntable ladder use, there was always a significant difference in satisfaction between animax mono and the electrically operated devices.

**Table 4:** Subjective satisfaction and physical burden (BORG scale) when using the various mCPR devices; *p*-values from the Kruskal-Wallis tests for each category

	Satisfaction when carrying (without turntable ladder operator)	Satisfaction when loading/unloading ambulance (without turntable ladder operator)	Satisfaction when using the turntable ladder (all)	Overall satisfaction (without turntable ladder operator)	Physical burden (BORG scale; without turntable ladder operator)
animax mono	3.5 (IQR 4)	5.0 (IQR 4)	4.0 (IQR 8)	5.0 (IQR 5)	4.5 (IQR 3)
AutoPulse	9.0 (IQR 2)	9.0 (IQR 2)	10.0 (IQR 2)	9.0 (IQR 1)	4.0 (IQR 3)
corpuls cpr	9.5 (IQR 2)	9.0 (IQR 1)	9.0 (IQR 2)	9.0 (IQR 1)	4.0 (IQR 3)
LUCAS 2	9.0 (IQR 3)	10.0 (IQR 2)	10.0 (IQR 1)	9.5 (IQR 2)	4.0 (IQR 2)
*p*-value (Kuskal-Wallis test)	0.001	0.002	0.010	0.001	0.754

**Table 5:** *P*-Values from post-hoc pairwaise comparsion between different devices using Bonferroni correction for multiple testing

Pairwise Comparison (Device 1 vs. Device 2)	Satisfaction when carrying (without turntable ladder operator)	Satisfaction when loading/unloading ambulance (without turntable ladder operator)	Satisfaction when using the turntable ladder (all)	Overall satisfaction (without turntable ladder operator)
animax mono – AutoPulse	0.016	0.049	0.083	0.015
animax mono – LUCAS2	0.016	0.004	0.011	0.001
animax mono – corpulse cpr	0.016	0.002	0.034	0.029
AutoPulse – LUCAS2	0.328	1.000	1.000	1.000
AutoPulse – corpuls cpr	0.126	1.000	1.000	1.000
LUCAS2 – corpulse cpr	1.000	1.000	1.000	1.000

## Discussion

In our study, the mCRP devices examined all yielded good results with respect to effective chest compression during pre-hospital patient transport. This observation is consistent with other studies that found higher quality of chest compressions with mCPR as compared to manual CPR when walking on a horizontal plane and on stairs or during ambulance transport and braking manoeuvres [17, 18]. Lyon et al. showed that Autopulse performed well when used during transport with soft stretcher [16]. Construction-design-related differences in stability were found, but did not lead to any clinically relevant worsening of chest compression parameters.

Over the course of the entire test, only once did two false pressure points occur at beginning of *soft stretcher* corpuls cpr-transport. However, since the connection point immediately before the two compressions registered as “incorrect” was checked and found to be correct for the subsequent stages, the number of incorrect pressure points of the mCPR devices was low or equal to 0, as in other studies [12, 14, 18].

Nevertheless, displacement of the connection point to the patient was most pronounced with animax mono. During transport with the *gurney*, the pressure point shifted by up to 4.5 cm, while no shifting was observed with all other devices during this stage. Shear and tensile forces on the control lever, which can be turned in all directions, are felt to be the cause, as they promote slippage at the connection point; in contrast to the results of Gaessler et al., in our study this did not lead to an incorrect pressure point [18]. The electrically powered mCPR devices seemed to be less susceptible to external forces by using a LDB (AutoPulse), spineboard (corpuls cpr) or stabilisation belts (LUCAS2).

During the tests, care was always taken to ensure that the mannequin was correctly secured on the gurney, but the manufacturer’s precautions (e.g. operate the device only when it is in a secure position) during transport were deliberately disregarded in order not to unnecessarily complicate analysis of the basic data and to reflect realistic use [24, 25]. Despite more or less marked instability, the devices had only a very low risk of slipping in such a way that the correct pressure point would have been lost, from which it can be deduced that regular checks of the compression point are necessary when using mCPR under transport. If this is ensured, then correct cardiac massage should be possible with all devices tested even under transport conditions.

With the manually operated animax mono, the percentage of compressions that were too deep when used in the *rescue basket* stage (40.1%) was noticeably high although a mechanical resistance in the device should prevent too deep compressions. One explanation for this observation could be, as with displacements, shear forces at the compression point. In the case of electrically powered mCPR devices, adjustments to the devices via automated calibrations may have played a role with respect to better values during transport: If the compressing agents were paused between stages in order to check the compression point, this could have led to better adaptation to the mannequin. In contrast to the study by Fox et al., the study by Gaessler et al. did not show any compression with a pressure depth that was in line with guidelines [14, 18]. The mannequin selected by Gaessler et al. [12, 18] could only inadequately represent the dimensions of a human thorax, whereas the mannequin used in our tests seems to be more suitable. According to the manufacturer, corpuls cpr adjusts to the elasticity of the thorax. Use on a mannequin might have led to incorrect pressure depth and thus cannot be transferred to humans. This may also explain the greater scattering in pressure depth observed during *basic resuscitation*.

Animax mono was subject to fluctuations in frequency and compression-ventilation ratio compared to electrically powered mCPR devices. These were most likely due to the manual operation and related transport influences. More than half of all stages were classified as “Not OK” with respect to the “30:2” compression-ventilation ratio. Measurement of compression frequency revealed that animax mono ranged from 88 to 112 compressions/minute; however, with a median of 100/min (IQR 9), this value was within the recommended range of 100–120/min) [21]. Gaessler et al. made similar observations [12, 14, 18]. Pauses ventilation could indicated by an acoustic and/or optical signal. Sunde et al. showed that this makes it easier to maintain the correct compression-ventilation ratio [17].

In all “satisfaction” categories, medians were at least 9.0 for electrically powered devices. Animax mono received significantly lower values ranging from 3.5 (“satisfaction when carrying”) to 5.0 (“satisfaction in loading/unloading the ambulance” or “overall satisfaction”). The similarly good performance of all devices for the category “physical burden” was surprising. Obviously, the control lever of animax mono minimized work so much that despite the long muscle-based operation, virtually no increased physical burden was perceived. Overall, participants rated the entire transport on the modified BORG scale (up to 10) as “somewhat/reasonably strenuous”. In the study by Fox et al., however, rescuers rated just an eight-minute manual chest compression on the BORG scale (RPE scale; values: 6–20) as “somewhat strenuous” (mean 13.6) [14]; Animax mono’s independence from a battery received not only praise but also disadvantages during transport. An assistant had to operate the device continuously and can not perform other activities. Apparently, this poses a tactical disadvantage over all electrically operated devices because they enable EMS personal to perform other activities und thus spare set of hands on-scene. Animax mono does not provide that. AutoPulse was praised for its “flat” design. However, study participants expressed criticism of the large back plate, which led to obstacles when laying the mannequin on the ambulance gurney. For corpuls cpr, participants evaluated the possible combination of a resuscitation arm with a spineboard very differently: immobilization was praised, while the effort required was viewed negatively. LUCAS2 was praised for its simplicity.

A primary limitation of the study is that the mannequin chosen does not allow assessment of blood flow to brain and coronary vessels. Physiological parameters - for ventilation as well - for the assessment of compression quality using mCPR during transport could not be verified. Furthermore, it was shown that resuscitation mannequins could influence the results because their biomechanical properties do not adequately represent the human body and the built-in measuring devices do not have the desired precision, at least for some of the parameters recorded. This study was purely descriptive. However, the small number of cases limits the informative value of the results.

## Conclusion

Along a transport route with typical obstacles such as stairs, turntable ladders or loading procedures and transport in an ambulance, all mCPR devices investigated in this study showed good to very good performance during transport under cardio-pulmonary resuscitation. Stability of the devices varied during transport, with no relevant incorrect pressure points observed. However, the results also show how important it is to regularly check stability and correct connection to the patient under transport conditions and to correct if necessary. However, when transferring the test to reality, losses in chest compression quality or injuries to the patient cannot be ruled out. Differences in the design of the devices were also reflected in the variable ratings by study participants. Interestingly, the use of animax mono, a purely muscle-powered device, did not mean higher physical burden. Automation seams to increase quality of resuscitation.

## Data Availability

The datasets used and/or analysed during the current study are available from the corresponding author on reasonable request.

## Abbreviations

ÄLRD: Ärztlicher Leiter Rettungsdienst
ca: Circa
cm: Zentimeter
CPR: Cardiopulmonary resuscitation
EMS: Emergency medical services
ERC: European Resuscitation Council
et al.: Et alii (and others)
GmbH: Gesellschaft mit beschränkter Haftung
Inc.: Incorporated
INM: Institut für Notfallmedizin und Medizinmanagement
IQR: Interquartile range
LUCAS: Lund University Cardiac Assist System
LMU: Ludwig-Maximilians-Universität München
mCPR: Mechanical cardiopulmonary resuscitation
p: Page
%: Percent
